# Overcoming Immunotherapy Resistance in Small-Cell Lung Cancer

**DOI:** 10.3390/biology15040356

**Published:** 2026-02-19

**Authors:** Matteo Canale, Fabrizia Suzzi, Alberto Verlicchi, Fabrizio Citarella, Angelo Delmonte, Paola Ulivi

**Affiliations:** 1 Biosciences Laboratory, IRCCS Istituto Romagnolo per lo Studio dei Tumori (IRST) “Dino Amadori”, 47014 Meldola, Italy; matteo.canale@irst.emr.it (M.C.); fabrizia.suzzi@irst.emr.it (F.S.); paola.ulivi@irst.emr.it (P.U.); 2 Department of Medical Oncology, IRCCS Istituto Romagnolo per lo Studio dei Tumori (IRST) “Dino Amadori”, 47014 Meldola, Italy; alberto.verlicchi@irst.emr.it (A.V.); angelo.delmonte@irst.emr.it (A.D.)

**Keywords:** small-cell lung cancer, immunotherapy, resistance mechanisms, bispecific antibodies, antibody–drug conjugates, adoptive cell therapies

## Abstract

Small-cell lung cancer (SCLC) is the most lethal histologic subtype of lung cancer. It is characterized by rapid tumor growth and early metastatic dissemination, resulting in an extremely poor prognosis. The addition of immunotherapy to standard chemotherapy has led to a modest improvement in survival for patients with extensive-stage disease. However, the overall clinical benefit remains limited due to both primary and acquired resistance to immunotherapy. Indeed, the efficacy of immune checkpoint inhibitors is influenced by multiple factors, including tumor heterogeneity, an immunosuppressive tumor microenvironment, and intrinsic molecular characteristics of the disease. In recent years, active research has been conducted to identify and therapeutically overcome resistance mechanisms to immunotherapy, and accumulating suggestive evidence is lighting the way for new strategies for clinical management of patients with SCLC. In this review article, we summarize and discuss the substantial obstacle to immunotherapy clinical efficacy, with a particular emphasis on the published and ongoing clinical trials that investigated the potential strategies to overcome mechanisms of resistance to immunotherapy. Moreover, we report and discuss the new therapeutic approaches tested, especially the use of antibody–drug conjugates, bi-specific antibodies, adoptive cell therapies and combination strategies.

## Simple Summary

Small-cell lung cancer is one of the deadliest malignancies worldwide. Resistance mechanisms to immunotherapy remain a limitation to clinical benefit. Combination therapies and innovative drugs are being tested, also in clinical trials, and new therapeutic strategies pave the way to overcome resistance to immunotherapy. In this review, we discuss preclinical and clinical evidence of these strategies.

## 1. Introduction

Lung cancer is the most common and the most lethal malignancy worldwide, accounting for almost 2.5 million new diagnosed cases and almost 2 million cancer-related deaths per year [1]. SCLC is a highly aggressive neuroendocrine malignancy, representing 13–15% of all lung cancers. It is characterized by rapid tumor growth and early metastatic dissemination, with an initially high sensitivity to both chemotherapy (CT) and radiotherapy (RT). Despite early responses, most patients relapse quickly, and long-term survival remains poor, with a 5-year survival rate below 7% [2,3]. The addition of immune checkpoint inhibitors (ICIs), particularly anti-PD-L1 agents combined with CT, has modestly improved outcomes in extensive-stage SCLC (ES-SCLC) [4,5]. In fact, a small subset of patients derives durable benefit, indicating the presence of intrinsic and acquired resistance mechanisms that limit the long-term efficacy of immunotherapy (IO). A key emerging concept in understanding this variability in treatment response is the molecular heterogeneity of SCLC. Recent transcriptomic analyses have defined distinct molecular subtypes of SCLC based on the expression of lineage-defining transcription factors: ASCL1 (SCLC-A), NEUROD1 (SCLC-N), POU2F3 (SCLC-P), and an inflamed subtype (SCLC-I) lacking neuroendocrine features and associated with immune infiltration [6,7]. These subtypes differ not only in their biology and oncogenic programs but also in their interaction with the immune system. Notably, the SCLC-I subtype exhibits higher T-cell infiltration, increased expression of immune checkpoint molecules, and a more “inflamed” tumor microenvironment, suggesting a potentially greater sensitivity to immunotherapy [7].

In contrast, the neuroendocrine (NE)-high subtypes (SCLC-A and SCLC-N) typically present with “immune-cold” phenotypes, characterized by low major histocompatibility complex (MHC) class I expression, poor antigen presentation, and minimal immune cell infiltration [8]. Such differences in immunogenicity likely contribute to variable responses to ICIs and underscore the importance of molecular stratification in guiding therapeutic decisions. Moreover, cancer stem cells (CSCs) play a key role in stemness maintenance of the tumor and in adaptation of cancer cells to selective pressure induced by drugs, as well as in addressing the immunosuppressive tumor microenvironment.

### Scope and Approach

In the last years, the introduction of new IO-based approaches revolutionized clinical trials designing for SCLC, and both pre-clinical and clinical research is currently ongoing, investigating new therapeutic molecules and new targets. Moreover, biomarkers helping patient stratification are lacking, especially those identifying resistance to immunotherapy, providing insights on how to overcome them. Owing to the challenging clinical behavior of SCLC, considerable attention has been devoted to the analysis of upfront combination strategies.

In this review, we synthetize the molecular mechanisms underlying resistance mechanisms to IO in ES-SCLC, and we provide a comprehensive update on the therapeutic strategies to overcome these mechanisms, including new therapeutic approaches and combination therapies. We also summarized the published trials that investigated the potential strategies to overcome primary or acquired resistance of IO-based regimens. Moreover, a critical review of the clinical trials testing these approaches is provided, as well as an in-depth discussion addressing future pre-clinical and clinical research.

For this review, we selected peer-reviewed clinical trials, early-phase studies, and high-quality translational research in SCLC, emphasizing emerging therapies and mechanisms of resistance. English-language publications and ongoing trials listed in ClinicalTrials.gov were included, while review articles and meta-analyses were used to provide contextual background.

## 2. Resistance Mechanisms to Immunotherapy

### 2.1. Molecular Subtypes and Heterogeneity

Recent advances in transcriptomic and epigenomic profiling have redefined SCLC molecular profiling. Based on dominant expression of ASCL1, NEUROD1, POU2F3, or YAP1, four molecular subtypes were identified (SCLC-A, -N, -P, and -Y, respectively) [6]. More recently, the SCLC-I subtype, characterized by an inflamed gene signature, was introduced instead of SCLC-Y as the expression of YAP-1 does not exclusively define a molecular subtype [7]. These subtypes not only reflect distinct cellular origins and oncogenic dependencies but also exhibit substantial differences in immunogenicity and therapeutic vulnerability, as suggested primarily by transcriptomic profiling and translational correlative analyses rather than prospective subtype-stratified clinical trials.

SCLC molecular subtypes with NE features, i.e., SCLC-A and SCLC-N, usually display immune desert characteristics and are theoretically less responsive to IO-based treatments. On the other hand, SCLC-P and -I, which are associated with the non-NE phenotype, have immune properties and are more likely sensitive to immunotherapy [7,9]. The SCLC-I subtype has emerged as a potentially more immunotherapy-responsive subgroup, based on translational analyses showing enrichment in interferon signaling, antigen presentation pathways, and immune cell infiltration; however, this observation remains exploratory and has not been prospectively validated as a predictive biomarker in clinical trials [7].

Altogether, these findings underscore the clinical relevance of molecular subtyping in SCLC, not only as a prognostic factor but also as a potential predictive biomarker for immunotherapy benefit.

Biological vulnerabilities and subtype-informed hypotheses are resumed in Table 1.

### 2.2. Low Tumor Immunogenicity

Tumor mutation burden (TMB) is defined as the number of somatic mutations per megabase and is generally considered a surrogate for tumor immunogenicity as a higher number of genetic mutations generally reflect increased neo-antigen productions [9]. SCLC is often referred as a high-TMB cancer, a feature typically associated with better response to immunotherapy [10,11]. However, SCLC generally shows limited and short-lived responses to both chemotherapy and immunotherapy, and TMB failed its predictive potential in both CASPIAN and IMpower133 trials [4,12]. A proposed explanation for the limited functional immunogenicity of SCLC neoantigens derives from impaired MHC class I-mediated antigen presentation, as supported by translational correlative studies in human tumor samples and preclinical models and also in SCLC [13,14,15]. In SCLC patients, this was confirmed by Mahadevan and colleagues, who found that a high proportion of SCLC has low MHC I expression; interestingly, MHC I high subpopulations displayed durable response to immunotherapy and were associated to the non-NE phenotype [16]. The relation between SCLC subtypes and MHC I expression was also highlighted by Gay and colleagues, who found that the SCLC-I subtype expressed high levels of immune and inflammatory genes, including programmed death-ligand 1 (PD-L1) and MHC-I, in spite of ASCL1, NEUROD1, and POU2F3 [7]. Moreover, the DNA damage response (DDR)–high phenotype, which is frequently observed in NE subtypes, has been associated with low MHC class I expression and minimal immune infiltration, thereby reinforcing an immune-evasive state and contributing to resistance to ICIs [17]. Another study found in 286 patients enrolled in the CheckMate032 that high expression of pathways related to antigen presentation was associated to long-term benefit [18].

Preclinical and translational evidence indicates that epigenetic regulators, including enhancer of zeste homolog 2 (EZH2) and lysine-specific demethylase 1 (LSD1), can suppress MHC class I expression in SCLC cells, thereby impairing antigen presentation. Clinical validation of these mechanisms as predictive biomarkers is currently lacking [16,19,20].

Even though accompanied by a minor interest, MHC-II has a potential in predicting response to immunotherapy, as highlighted by an exploratory analysis conducted on patients enrolled in the CASPIAN trial, which demonstrated longer OS in the durvalumab and tremelimumab plus chemo-etoposide treated patients with human leukocyte antigen (HLA) class II allele DQB1*03:01 [21].

### 2.3. Immunosuppressive Tumor Microenvironment

The tumor microenvironment (TME) is composed of a structural dynamic complexity of tumor cells and surrounding non-cancerous host cells including different subpopulations, such as immune regulatory T (Tregs), tumor-associated macrophages (TAMs), and myeloid-derived suppressor cells (MDSCs), as well as stromal cells (e.g., fibroblast and endothelial cells). These cells provide a highly structured environment that sustains cancer growth and metastasis, also producing cytokines and metabolites that are able to influence each other to create a pro-tumorigenic environment [22]. The low response rate of SCLC immunotherapy may be associated with the poor number of immune cells infiltrating both the tumor immune microenvironment and the tumor nest [23].

Natural killer (NK) cells are a key population of tumor-infiltrating lymphocytes, representing an important element of host antitumor immunity. Preclinical models have demonstrated that NK cell depletion significantly increases the risk of SCLC metastasis, supporting a potential role for NK cell activation strategies; consequently, adoptive cellular therapies represent a conceptually attractive but still experimental approach, with evidence largely restricted to preclinical models and early-phase clinical experience in highly selected patients [24].

Tumor immune lymphocytes (TILs) CD8+ are the key immune effectors against tumor cells and are essential for the therapeutic efficacy of immunotherapy as they represent the main target of ICIs [25]. At this regard, SCLC is considered an “immune-cold” tumor as TILs are significantly lower with respect to non-small-cell lung cancer (NSCLC) [26].

Tregs are the most represented immunosuppressive cells in the TME, by secreting TGF-β and IL-10 and expressing CTLA-4 and PD-1, more abundant in advanced SCLC stages and associated with worse prognosis [27,28,29]. However, evidence regarding their prognostic impact derives from retrospective and correlative studies and remains inconsistent across cohorts. On the other hand, it has been found that high intra-tumoral infiltration of FOXP3+ Tregs predicted favorable outcomes (*p* = 0.006), while another study found that patients with SCLC with high FOXP3 expression had longer time to relapse compared with patients with low FOXP3 expression [30,31].

MDSCs act as a key negative regulator of CD8+ TILs; they represent a heterogeneous subpopulation of myeloid cells with high immunosuppressive potential and are associated with advanced stage and short survival of patients with SCLC [32]. Interestingly, MDSCs have been explored as a therapeutic target primarily in preclinical models and early translational studies, with limited clinical evidence to date. All-trans retinoic acid (ATRA) has the capability of inducing MDSC differentiation in dendritic cells and macrophages [33]. It has been observed that gemcitabine plus a checkpoint kinase 1 inhibitor reduce MDSCs production, together with a lowering of M2-type TAMs and Tregs in the TME [34]. A case of a patient with refractory SCLC treated with combination nivolumab and temozolomide was reported, experiencing a durable clinical response accompanied by an early and stable decrease of MDSCs and improved T CD8+ cell function [35].

TAMs are a plastic immune cell population that can differentiate in the M1 (pro-inflammatory) or M2 (anti-inflammatory) phenotype, depending on the different stimuli they receive from the tumor and immune microenvironment. M2-phenotype macrophages are able to directly suppress T-cell recruitment and function, regulating tumor immunity and representing a substantial obstacle to immunotherapy efficacy [36]. M2-TAMs are extensively represented in the SCLC TME, suggesting their direct involvement in SCLC progression [37]. Even though several preclinical attempts have been performed, the efficacy of TAMs targeting as a therapeutic approach reached few suggestive clinical results.

In the field of cancer IO, the introduction of ICIs provided effective results in different solid malignancies. One of the resistance mechanisms of SCLC to such drugs is the redundancy of the immune checkpoints in the TME and activation of alternative immune inhibitory pathways to compensate the blockade of immunosuppressive ligands, which leads to inconsistency of ICIs [38]. To date, several alternative checkpoint activation pathways have been highlighted in cancer, including upregulation of TIM-3, LAG-3, TIGIT and VISTA, which often share downstream signaling cascades with CTLA-4 and PD-1/PD-L1 [39,40,41,42,43].

The concomitant blockade of different checkpoint inhibitors is a suggestive clinical option that is currently under investigation.

### 2.4. Role of Cancer Stem Cells

Over the past decade, the hypothesis that CSCs play a crucial role in tumor relapse and drug resistance has gained great attention in cancer clinical research. CSCs represent a rare subset of tumor cells characterized by properties such as stemness, self-renewal, and multilineage differentiation. In the context of SCLC, CSCs have been implicated in tumorigenesis, treatment resistance, and metastatic potential in SCLC, based largely on preclinical and translational studies, mainly through investigation of mechanisms such as the epithelial–mesenchymal transition (EMT) [44].

CT and RT treatments can kill most of the highly differentiated SCLC cells, leading to rapid tumor shrinkage. However, residual CSC fractions (CD133+) are not effectively eradicated, representing a potential drive of tumor recurrence [45].

CSCs showed a high level of multidrug efflux pumps reducing intra-cellular concentration through enhanced cellular efflux [46].

Preclinical studies have shown that both tumor cells and CSCs can modulate antitumor immunity by expressing immune checkpoint molecules or by secreting various soluble factors promoting immune evasion [47]. According to Mansour et al., PD-L1 expression on CSC on breast cancer cells was three times higher than that observed in differentiated cells [48]. In line with these findings, Kursunel et al. reported high PD-L1 expression in a cancer stem sub-population CD44^+^CD90^+^ isolated from primary SCLC samples [49].

Wei et al. demonstrated that PD-L1 helps CSC to keep their stem-like properties in colorectal cancer by activating High-Mobility Group AT-hook 1 (HMGA1) signaling pathways; notably, when HMGA1 was blocked, the ability of PD-L1 to support stem cell traits was limited [50].

In SCLC, factors including IFN-γ and IL-8 have been reported to drive PD-L1 upregulation in CSCs [51]. Fu et al. reported that higher circulating levels of TGF-β in SCLC patients were linked to worse patients’ survival, as well as a higher number of CD133^+^ CSCs. Using a TGF-β inhibitor, namely, SB431542, the authors observed a reduced stem-like phenotype of these cancer cells [52]. Interestingly, CSCs can evade immune surveillance by downregulating MHC-I expression, and upregulation of MHC-I in CSCs through IFN-γ treatment would increase cells’ sensitivity to immunotherapy and prolong survival in mouse models [53]. Another attempt to target CSCs was performed by Best and colleagues, who found that boosting IL-15 or TGF-β signaling activates NK cells, increasing their ability to kill CSCs in SCLC, an effect that is even stronger when combined with an anti-PD-1 [24]. Moreover, CSCs can directly shape the immune TME by inducing the M2 macrophage phenotype, thus contributing to create an immunosuppressive TME [54].

**Figure 1:**
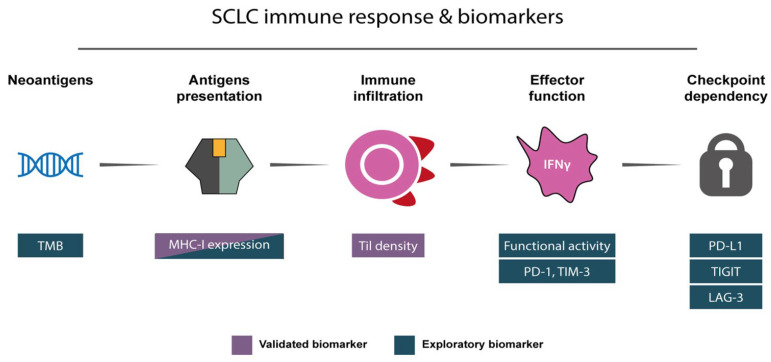
Conceptual framework linking tumor immunogenicity, antigen presentation, immune infiltration, effector function, and checkpoint dependency in SCLC. Blue and lilac boxes indicate each biomarker as a validated or an exploratory one.

A simplified conceptual framework resuming validated and exploratory biomarkers for immunotherapy in SCLC is presented in Figure 1. Biomarkers are contextualized throughout the manuscript to discuss the new therapeutic approaches.

Altogether, these findings suggest that the interaction between CSCs and macrophages creates a cycle that may reduce the effectiveness of immunotherapy in SCLC.

## 3. Strategies to Bypass SCLC Resistance to Immunotherapy

At the light of biological biomarkers and preclinical evidence highlighted to date, there is a growing interest in finding the best therapeutic strategy to overcome IO resistance mechanisms in SCLC. As SCLC is a heterogeneous malignancy with plastic cell phenotypes, the currently ongoing approaches focus on the targeting of different markers at the same time, to help host immune system to kill cancer cells, also creating physical bridges between effectors CD8+ T and cancer cells.

**Figure 2:**
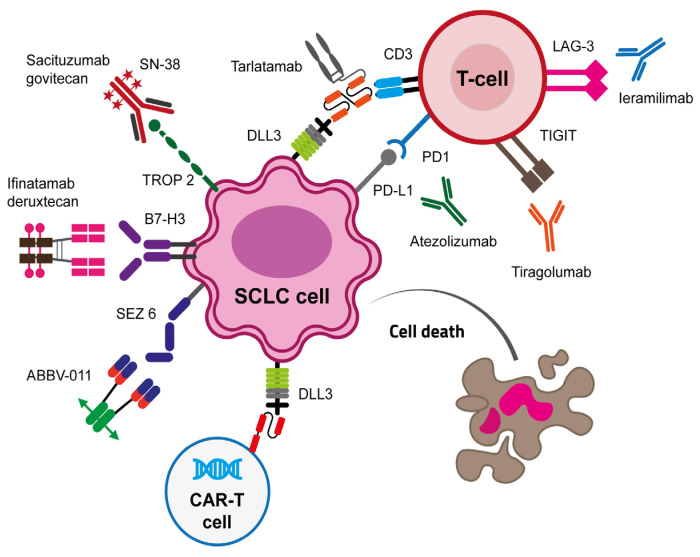
Novel approaches to overcome resistance to immunotherapy in small-cell lung cancer.

In the next paragraph, we discuss the latest approaches that reached the best clinical performance, and we provide an exhaustive view of the new approaches and strategies under investigation to date (Figure 2).

### 3.1. Bispecific Antibodies

Delta-like ligand 3 (DLL3) is an inhibitor of the Notch signaling, which can promote SCLC cell proliferation in vivo via Snail1 activation. It often shows overexpression in SCLC, especially in SCLC with a marked neuroendocrine phenotype, i.e., SCLC-A and SCLC-N subtypes [55,56,57,58]. In a study considering a large case series of 1073 SCLC patients, DLL3 expression (defined as ≥25% of positive tumor cells) has been found in 85% of patients, while 68% of patients had a high expression of DLL3 (defined as ≥75% of positive tumor cells) [59]. Importantly, despite DLL3 expressing tumors having a higher burden of neo-antigens, they display a suppressed tumor immunity by inhibiting antigen presenting dendritic and macrophage cells. The effect is predominant also compared with low DLL3 tumors, suggesting that DLL3 expression contributes to ICIs resistance [60]. Its high expression in SCLC and minimal expression in normal tissues attracted the attention for precision medicine. The first attempt to target DLL3 was performed by the rovalpituzumab terisine (Rova-T) antibody–drug conjugate (ADC), which did not demonstrate clinical efficacy in phase II TRINITY trial [61].

The first-in-human bispecific antibody BI 764532 was evaluated in a phase I dose-escalation study (NCT04429087) involving 90 patients with neuroendocrine carcinoma (48%) and SCLC (52%). The median treatment duration was 43 days (range: 1–443). Treatment emergent adverse events (TEAEs) occurred in 86% of patients, with the most frequent grade ≥ 3 event being a decrease in lymphocytes (14%). The maximum tolerated dose was not reached. Among the 24 SCLC patients who received at least the target dose of the bispecific T-cell engager (BiTe), the objective response rate (ORR) was 33% by the investigators’ assessment [62,63].

Immunotherapeutic targeting of DLL3 demonstrated clinical benefit as a further line of treatment for patients with SCLC, through the first-in-class BiTe, namely, tarlatamab, which received the FDA approval in May 2024, based on the results of the phase II DeLLphi-301 trial, in which ORR was evaluated by blinded independent central review [64]. Bispecific antibodies, or BiTe molecules, are recombinant immunoglobulins consisting of two single chain variable fragments (scFv) connected in tandem by a flexible linker, able to target two targets together, a tumor-associated antigen and a T-cell antigen, usually CD3. These molecules exert their activity by circumventing MHC-I/T-cell receptor (TCR) engagement through the establishment of a physical bridge between T cells and tumor cells, thereby inducing T-cell activation, tumor cell cytotoxicity, and cytokine release via cytolytic immunological synapses, bypassing defects in antigen presentation and immune infiltration presented in Figure 1 [65].

Results from the phase III DeLLphi-304 trial have been recently published; tarlatamab demonstrated a practice-changing benefit in investigator-assessed both progression-free survival (PFS) and overall survival (OS) versus standard treatment as a second-line treatment in SCLC patients whose disease had progressed to a platinum-based CT (median OS (mOS) 13.6 months, 95%CI: 11.1-NR vs. mOS 8.3 months, 95%CI: 7.0–10.2, respectively; median PFS (mPFS) 4.2 months, 95%CI 3.4–4.5, vs. 3.7 months, 95%CI 2.9–4.2, respectively). The study enrolled 509 patients, of which 71% received a PD-1 or a PD-L1 inhibitor in combination with CT as a first-line treatment. Interestingly, in the subgroup analysis, tarlatamab conferred a better prognosis in terms of both OS and PFS in IO-treated patients, with respect to patients treated with solely CT (HR_OS_ 0.61, 95%CI: 0.45–0.82 vs. HR_OS_ 0.65, 95%CI: 0.42–1.03, respectively; HR_PFS_ 0.67, 95%CI: 0.53–0.84 vs. HR_PFS_ 0.89, 95%CI: 0.62–1.28, respectively) [66].

The results for the main clinical trials investigating new possible therapeutic approaches as a first-line treatment are reported in Table 2. Considering higher expression of DLL3 in NE phenotype and a higher expression of MHC-I in non-neuroendocrine phenotype [16,58], BiTe could have a synergistic effect with canonical anti-PD-1/PD-L1 inhibitors, which need MHC-I/TCR for T-cell activation, especially for mixed phenotype neoplasms.

DLL3 targeting was also performed using tri-specific T-cells activating constructs (TriTACs), T-cell engagers which can additionally bind human serum albumin [67]. HPN328 is being tested in a phase I/II trial enrolling patients with high-grade NE tumors with DLL3 expression, including SCLC, alone or in combination with atezolizumab, or in combination with the ADC ifinatamab–deruxtecan (NCT04471727). Encouraging preliminary results have been reported, demonstrating an investigator-assessed ORR 37% and a disease control rate (DCR) of 78% for patients with brain metastases and an ORR of 19% and a DCR of 48% without brain metastases [68].

Even though DLL3 targeting is demonstrating clinical benefit for patients, resistance mechanisms occur in most cases. In particular, different studies found that on-target resistance mechanisms, such as Notch pathway alterations, are associated with resistance to DLL3 targeting [69].

Lymphocyte activation gene-3 (LAG-3) is a transmembrane protein that plays a key role in T-cell proliferation and cytolytic functions [70]. Lag-3 is expressed on activated CD4+ and CD8+, and it can negatively regulate expansion of T cells by interfering with CD4 binding to MHC-II [71,72]. A phase I trial investigated the maximum tolerated dose alone and in combination with pembrolizumab of an anti-CTLA4 anti-LAG-3 BiTe, namely, XmAb^®^22 841, and results are awaited (NCT03849469).

The targeting of neo-angiogenesis is a therapeutic strategy that has been attempted in different solid malignancies, resulting in the approval of different compounds (e.g., sunitinib, sorafenib, and ramucirumab) for advanced diseases [73]. In SCLC, the combination of carboplatin/etoposide with a bispecific antibody targeting PD-L1 and vascular endothelial growth factor (VEGF), namely, ivonescimab, was recently tested in a phase I trial enrolling 35 patients with ES-SCLC receiving 3 mg/kg, 10 mg/kg, or 20 mg/kg of the drug plus CT, with ivonescimab as maintenance. Global ORR and DCR were investigator-assessed as 80.0% and 91.4%, respectively (ORR 66.7%, 90.9% and 76.2% for the three doses’ treatment arms, respectively). Grade ≥3 TEAEs occurred in 60% of patients, with neutrophil decrease as the most common [74].

A similar bispecific antibody targeting PD-L1 and VEGF-A, namely, PM8002, was tested in a phase II trial in combination with paclitaxel in 27 pre-treated patients with SCLC, reaching an ORR and a DCR of 72.7% and 81.8%, respectively, with an mPFS of 5.5 months (95%CI: 2.8-NR). Grade ≥ 3 TEAEs occurred in 73.1% of patients, with the most common observed as neutropenia (53.8%) [75].

**Table 2:** Combination strategies in the first-line setting. Therapeutic strategies tested in ES-SCLC or LS-SCLC as a first-line therapy are presented. Treatment schedules, as well as primary and secondary endpoints and key study limitations, are highlighted. ES-SCLC: extensive-stage small-cell lung cancer; LS-SCLC: limited-stage small-cell lung cancer; EC: carboplatin/etoposide; mPFS: median progression-free survival; mOS: median overall survival; ORR: objective response rate; TRAEs: treatment-related adverse events.

Experimental Design	Phase	Disease Stage/Setting	Comparator	Primary Endpoints	Secondary Endpoints	Major Limitations	Ref
Entinostat + atezolizumab + carboplatin/etoposide	I	ES-SCLC. 3 patients	None	2 patients reporting DLTs at DL1.	Not explorable due to accrual stopping	Extremely small cohort; exploratory aims	[76]
Atezolizumab + EC ± tiragolumab	III	490 ES-SCLC	Placebo-controlled	mPFS: 5.4 vs. 5.6 months for tiragolumab and control arm. mOS:13.6 months for both arms.	ORR 70.8% vs. 65.6%; G3-4 TRAEs: 52.7% vs. 55.7% for tiragolumab and control arm, respectively.	Negative trial; no efficacy benefit	[77]
Benmelstobart (B) + anlotinib (A) + EC vs. controls (1:1:1)	III	ES-SCLC. 246 B + A + EC 245 A + EC 247 EC	Dual placebo arms	Prolonged mPFS and mOS for B + A + EC and A + EC vs. control. Prolonged mPFS for A + EC vs. control.	ORR 81.3% vs. 66.8% for B + A + EC and EC (*p* = 0.0001). ORR 81.2% vs. 66.8% for A + EC vs. EC (*p* = 0.0003). G ≥ 3 93.1%, 94.3% and 87% for B + A + EC vs. control, respectively.	Complex design; regional population	[78]
Surufatinib + toripalimab + cisplatin/etoposide	IB/II	LS/ES- SCLC. 35 patients	None	mPFS 6.9 months (6-month PFS rate 50.44%, 12-month PFS rate 27.69%).	ORR 97.1%; DCR 100%; mDOR 5 months. mOS 21.1 months (12-month OS rate 66.94%).	Non-randomized; mixed stages	[79]
Ivonescimab (dose escalation) + carboplatin/etoposide (3 mg/kg: A, 10 mg/kg: B or 20 mg/kg: C) + EC	IB	ES-SCLC. Ivonescimab (3 patients 3 mg/kg: A, 11 patients 10 mg/kg: B, 21 patients 20 mg/kg: C)	None	G ≥ 3 TRAEs: 66.7%, 54.5% and 61.9% for A, B, C arms ORR 66.7%, 90.9% and 76.2% for A, B, C.	DCR 91.4%. mDOR 5.6 months. mPFS 6.9 months (NR, 7, and 6.9 months for A, B, C). mOS 14.5 months (NR, 17.1, and 14.5 months for A, B, C).	Phase I with small cohort; dose finding	[74]
Atezolizumab + bevacizumab + EC	II	ES-SCLC. 53 patients	None	1 year OS rate 61.8%. mOS 12.9 months.	ORR 83.3%. mPFS 6.2 months. G3-4 AEs 64.2%, 4 cases G5. Treatment-related SAEs 35.8%.	Single-arm; no control	[80]

**Toxicity and feasibility considerations:** Across DLL3-targeting bispecific antibodies, toxicity is largely driven by on-target T-cell activation. Cytokine release syndrome (CRS), predominantly grade 1–2, represents the most characteristic class-specific adverse event, with grade ≥ 3 events being uncommon but requiring careful monitoring and step-up dosing strategies. Transient cytopenias, particularly lymphopenia, have been reported as dose-limiting toxicities in early-phase trials. Treatment discontinuation due to adverse events remains relatively infrequent, supporting feasibility in pretreated populations; however, CRS risk and immune-mediated toxicities may limit combination with other immune-activating agents or require careful sequencing rather than concurrent administration.

#### Clinical Positioning and Therapeutic Sequencing Considerations and Key Uncertainties

As a second-line setting, DLL3-targeting bi-specific T-cell engagers currently represent the most clinically advanced immune-redirecting strategy for ES-SCLC. Moreover, their mechanism of action by directly engaging CD3+ cells provides a strong biological rationale related to the disease. From a clinical pragmatic point of view, bispecific antibodies may be preferentially considered in patients with slower disease kinetics and preserved performance status, including those previously exposed to ICIs, as supported by the DeLLphi-304 trial. Despite these encouraging results, generalizability remains limited: the efficacy in underrepresented subgroups (patients with active brain metastases, ECOG PS > 1, frail or comorbid patients) is unclear. Differences in comparator regimens (various standard chemo schedules) may affect the observed effect size. Moreover, predictive biomarkers (e.g., DLL3 expression intensity) for response remain undefined, as well as long-term quality of life and tolerability in real-world populations.

### 3.2. Antibody–Drug Conjugates

ADCs are composed of a tumor targeting monoclonal antibody (mAb) conjugated to a cytotoxic payload through a chemical linker. These molecules are able to precisely target a tumor antigen and to potently act as cell killing agents via cytotoxic payload, also resulting in a bystander effect on neighboring cells [81].

B7 homolog 3 (B7H3), also named CD276, plays a key role for immune suppression in cancer progression [82]. Its overexpression in solid tumors, including SCLC, and its role in tumor growth, metastasis and resistance to therapy led to several attempts of therapeutic targeting of B7H3 [26,83].

Ifinatamab–deruxtecan (I-DXd) uses the topoisomerase I inhibitor deruxtecan as a cytotoxic payload. The single-arm phase II trial IDeate-Lung01 established the dose of 12 mg/kg as the optimal dose by blinded independent central review per RECIST 1.1, for the extension of the ongoing phase III IDeate-Lung02 trial (NCT06203210), considering both an almost double ORR compared with the 8 mg/kg (52.4% vs. 26.1%, respectively) and a higher number of adverse events (AEs), mostly hematological and gastrointestinal. Even though not significant, PFS and OS showed a trend in favor of the 12 mg/kg dose at interim analysis [84].

Based on the results from NCT04145622, the phase I/II trial investigated the clinical efficacy/safety of DS-7300 in 127 patients with solid tumors (9 SCLC) in 4.8–16.0 mg/kg cohorts. In this group, DCR by investigators’ assessment was 77.8%, with observed grade ≥3 treatment-related adverse events (TRAEs) more frequently occurring in the 16 mg/kg treated cohort with respect to cohorts treated with 8.0 and 12.0 mg/kg [85].

A similar ADC, carrying a topoisomerase inhibitor, is HS-20093, the clinical efficacy of which was tested in the ARTEMIS-001 phase I trial. Also in this study, two doses of 8 mg/kg and 10 mg/kg were investigated, with favorable data for the higher dose in terms of DCR (81% vs. 96%, respectively) and mPFS (5.9 vs. 7.3 months, respectively) [86].

Recent results for a phase I/Ib trial testing the YL201 ADC enrolling 287 patients have been reported, which is composed of a topoisomerase 1 payload in a drug-to-antibody ratio of 8, with a dual cleavage and release mechanism, as the complex composed of the antigen and the ADC is internalized by lysosomal vesicles and the toxic payload is released, leading to apoptosis activation and bystander effect [87]. In the group of 68 patients with ES-SCLC, ORR was 63.9% (95%CI: 51.7–74.9), and the DCR was 91.7% (95%CI: 82.7–96.9), with an mPFS of 6.3 months (95%CI: 5.6–7.6). In the five patients who received a prior topoisomerase 1 inhibitor (topotecan or irinotecan), ORR was lower at 20%, suggesting the existence of resistance mechanisms to such agents. The dose expansion involved a 2.4 mg/kg^−1^ and a 2.0 mg/kg^−1^ treatment group; TEAEs were mostly hematological, with a grade 3 incidence of 71.1% and 50.0%, respectively; ORR was similar in the two groups (63.9% and 67.7%), while mPFS values by dose were 5.7 and 7.6 months, respectively. No correlation was observed between B7H3 expression and clinical response, suggesting that B7H3 expression could not be considered as a predictive biomarker [87].

Seizure-related homolog 6 (SEZ6) is a transmembrane protein found at high expression in SCLC cells, with respect to a low expression in normal tissues, and identified as a downstream target of ASCL1 [6,88]. The SEZ6 targeting mAb SC17 conjugated with calicheamicin, a DNA-damaging agent used as a payload, namely, ABBV-011, was tested in a phase I trial (NCT03639194), with dose escalation of 0.3–2 mg/kg and expansion of 1.0 mg/kg for selected SEZ-6 expression-positive patients (≥25% of positive tumor cells by IHC). Ninety-nine pre-treated patients received ABBV-011 monotherapy, with a global ORR of 19%, while in the expansion cohort, ORR was 25%, with an mPFS of 3.5 months. Grade 5 TEAEs were reported in 19% of patients who received at least one dose of ADC, while the most common grade 3 TEAEs were hepatotoxicity and fatigue (12% and 9%, respectively). Notably, the initial expansion cohort dose of 2.0 mg/kg was reduced to 1.0 mg/kg due to multiple hepatotoxic TEAEs, indicating that the optimal dose remains to be determined.

Trophoblast cell surface antigen 2 (Trop-2) overexpression has been associated with poor survival in different solid malignancies, often co-occurring with pathogenic gene mutations (e.g., *TP53* in breast, colorectal, pancreatic and hepatocellular carcinomas), a more active immune tumor microenvironment, suggesting clinical benefit of Trop-2 targeting in combination with immunotherapeutic agents [89,90,91].

Sacituzumab govitecan (SG) conjugates an anti Trop-2 Ab to the irinotecan metabolite SN-38 and is approved for the treatment of triple-negative pre-treated patients with breast cancer [92]. The phase II TROPiCS-03 (NCT03964727) enrolled 43 pre-treated ES-SCLC, reporting an ORR of 41.9% (95%CI: 27.0–57.9%), while mPFS and mOS were 4.7 (3.5–6.7) and 4.4 (3.8–6.1) months, respectively. TEAEs of grade ≥ 3 were reported in 74.4% of patients, with serious AEs in 51%, with febrile neutropenia as the most common (7.0%) [93].

The topoisomerase inhibitor payload has been used for anti-Trop-2 SHR-A1921 ADC construct; a phase I study in pre-treated patients reported an ORR and a DCR of 33.3% (95%CI: 15.2–58.3) and 66.7% (95%CI: 41.7–84.8), respectively, with an mPFS of 3.8 months (95%CI: 1.4-NR). Notably, all evaluable patients for clinical endpoints had low expression of Trop-2 [94].

**Toxicity and feasibility considerations:** ADCs in SCLC are characterized by a toxicity profile largely driven by payload-related effects rather than target expression. Hematologic toxicities, including neutropenia, anemia, and thrombocytopenia, are frequent and represent the main cause of dose reductions and treatment delays. Gastrointestinal toxicity and fatigue are commonly reported, while hepatotoxicity has emerged as a relevant safety concern for selected constructs, leading to dose de-escalation in expansion cohorts. Discontinuation rates due to toxicity are non-negligible, particularly at higher doses, and cumulative myelosuppression may limit sequential use after prior topoisomerase-based chemotherapy or combination with cytotoxic regimens.

#### Clinical Positioning and Therapeutic Sequencing Considerations

From a mechanistic point of view, ADCs offer a different approach in relapsed ES-SCLC as these compounds are able to deliver potent cytotoxic payloads directly to the tumor cells, independently of the immune engagement and from the immune cascade proposed in Figure 1. This could be particularly attractive in patients with rapidly progressive disease or high tumor burden, where immediate tumor control is clinically prioritized.

Despite encouraging response rates across multiple targets, including B7-H3, SEZ6 and Trop-2, clinical benefits have been not durable, and treatment is often associated with hematologic and gastrointestinal toxicities. From a clinical point of view, these treatments could have a potential when immune-based strategies are not effective. Moreover, the evidence is not strong, as it mostly results from early-phase and single-arm studies, and no ADC has been validated in randomized phase III trials versus standard of care, limiting direct comparisons on OS or other clinically meaningful endpoints.

### 3.3. Adoptive Cell Therapies

Chimeric antigen receptor (CAR) is becoming a common strategy to fight cancer by engineering immune cells receptors on patient-derived immune cells to bind a tumor antigen and proliferate against tumor cells; these approaches include CAR-T cells, TILs, TCR-engineered cells, and CAR-NK cells; engineered receptors are composed of an extracellular scFv, a transmembrane spacer, and an intracellular domain [95]. Considering that SCLC lacks canonical antigen presentation, a CAR-based approach could be a bridge between immunotherapy and targeted therapy.

SCLCs are often characterized by the loss of MHC-I and the limitation of gathering a fully representative tumor sample, and the TCR-T and TIL approaches have limited possibilities in SCLC, while the CAR-T technique results in the most promising adoptive cell therapy approach [96]. Similar to other oncologic biomarkers, ideal targets for CAR therapies are those that are uniformly and highly expressed on tumor cells, with limited or no expression on normal tissues. Therefore, most of the clinical research based on CAR approaches focused on DLL3 targeting.

In SCLC preclinical models, DLL3 CAR-T inhibited cancer growth without damaging normal cells, and another study observed that DLL3 CAR-T secreted pro-inflammatory IL-18 contributed to long-term response [97,98].

AMG119 contains a DLL3-targeting CAR, enhancing T-cell-mediated response against DLL3-expressing cells. After the significant anti-tumor activity in-vivo, a phase I study (NCT03392064) tested safety and tolerability of AMG119 in SCLC relapsed patients, with 1/5 participants experiencing a grade 3 TEAE; one patient of the 4 clinically evaluable patients achieved a partial response; and the trial was suspended for nondisclosed reasons (NCT03392064) [99]. Other currently ongoing phase I trials investigating the potential of CAR-T cells in SCLC are using LB2102, SNC-115, and BHP01 [NCT05680922, NCT06384482, and NCT06348797].

Moreover, DLL3 targeting through ALLO-1213 showed therapeutic potential in preclinical studies to evaluate the therapeutic potential, and clinical testing is awaited [97].

AC133, also known as CD133, has been investigated as a potential target for CAR-T approaches. Preliminary results showed an inhibition of cancer growth and a synergistic effect with PD-1 blockade in mice with metastatic SCLC [100].

Disialoganglioside GD2 and Ganglioside GM2 have been found to be expressed in SCLC, thus representing a possible target for CAR-T therapies [101]; after encouraging preclinical results using induced pluripotent stem cells (iPSCs) to derive GD2 CAR-T cells, a phase I study evaluating dosing and clinical potential is currently ongoing (NCT05620342) [102].

DLL3 targeting has been also evaluated using a CAR-NK cell, specifically NK-92. Cytotoxicity and cytokine production were significantly highlighted by in vitro co-culturing of DLL3 CAR-NK-92 and DLL3+ SCLC cells, and in DLL3+ SCLC xenografts, CAR-NK-92 demonstrated significant tumor invasion [103]. These preclinical results led to the start of a phase I study enrolling relapsed or refractory extensive-stage SCLC (NCT05507593).

**Toxicity and feasibility considerations:** Adoptive cellular therapies in SCLC remain constrained by both safety and logistical challenges. While early-phase trials have reported manageable acute toxicities, including cytokine-mediated events and transient organ dysfunction, the limited number of treated patients precludes a comprehensive safety characterization. Moreover, manufacturing complexity, the need for specialized centers, and prolonged treatment timelines significantly limit real-world feasibility. These factors currently confine cellular therapies to highly selected patients within clinical trials rather than routine later-line sequencing strategies.

#### Clinical Positioning and Therapeutic Considerations

Even though CAR cells reached encouraging results, all evidence derives from pre-clinical studies, at an early-phase development. Enrollment in clinical trials is ongoing, and results will indicate the clinical utility and possible algorithm positioning. On the other hand, a key limitation of CAR cells is the need of a stable and uniformly expressed tumor target, which is rarely the case for SCLC.

### 3.4. Combination of Radiotherapy and Immunotherapy

In the context of ES-SCLC, radiotherapy is often performed with consolidation or prophylactic cranial intents after first-line therapy or with palliative aims, while for LS-SCLC it is the standard of care when combined with chemotherapeutic agents [104].

Given the peculiar sensitivity to radiation therapy, integrated strategies were explored combining with systemic treatment, beyond or before the canonical consolidation extent. Pembrolizumab was tested simultaneously with thoracic irradiation both in the limited and in the extensive settings. In absence of a control arm, the results were suggestive of benefit especially for the patients affected by limited disease, likely due to better prognosis of lower disease dissemination [105,106]. Larger trials are awaited to confirm the combination treatments’ efficacy.

Similarly, the upfront combination of thoracic low-dose radiotherapy and platinum-based chemotherapy plus atezolizumab showed a PFS rate at 1 year of 56.% and a consistent PFS of 6.9 months [107].

In a small trial comparing durvalumab plus tremelimumab with or without stereotactic body radiation therapy (SBRT), the intensive treatment schedule did not correlate with better outcomes [108]. The clinical trials investigating the addition of radiotherapy to immune checkpoint inhibitors are summarized in Table 3.

#### Clinical Positioning and Therapeutic Considerations

Radiotherapy retains an important role in ES-SCLC, especially for consolidation after systemic response or palliation in oligo-progressive sites. In the context of radio-immunotherapy, radiation may have immune-sensitizing effects, even though robust prospective data confirming survival benefits are limited. On the other hand, no phase III randomized trials have demonstrated significant survival improvement with systematic integration of RT and IO versus standard therapy, particularly in ES-SCLC.

### 3.5. Combination of Antiangiogenic Therapies

Combinations of antiangiogenic agents with IO have yielded high response rates in selected cohorts, but evidence is largely derived from non-comparative studies. The extent to which these responses translate into durable survival benefit remains unclear and requires randomized validation. Anlotinib with bortezomib, paclitaxel, and carboplatin plus etoposide ameliorated the progression-free survival, when administered with or without the anti-PD-L1 benmelstobart (mPFS 6.9 vs. 4.2 months for benmelstobart + anlotinib + etoposide/carboplatin vs. etoposide/carboplatin; *p* < 0.0001; median PFS 5.6 versus 4.2 months for anlotinib + etoposide/carboplatin versus 4.2 months, *p* < 0.0001). The benefit in terms of OS was statistically significant only in the anlotinib plus benmelstobart plus chemotherapy arm [78]. The outcomes reported by Lamberti and colleagues in a phase II study confirm the potentiality of anti-angiogenic integration strategies, with an OS rate at 1 year of 61.8% and an ORR of 83.3% [80].

Anti-angiogenic effects are included in the therapeutic spectrum of surufatinib, whose activity in association with the anti-PD-1 toripalimab was tested in a phase IB/II trial [79]. Despite the absence of a direct comparison with standard of care, the median PFS of 6.9 months, combined with an ORR of 97.1% and a DCR of 100%, builds a solid rationale for the design of future clinical trials.

Similar results derive from the administration of the anti-VEGF/PD-1 bi-specific antibody ivonescimab. Toxicity rates and clinical outcomes are not proportional to the dosages tested [74].

With regard to toxicity and feasibility considerations, anti-angiogenic agents combined with chemotherapy and/or immunotherapy introduce a distinct toxicity spectrum, including hypertension, proteinuria, hemorrhagic risk, and overlapping hematologic adverse events. Although generally manageable, these toxicities may complicate combination strategies in heavily pretreated or frail patients and require careful patient selection and monitoring.

#### Clinical Positioning and Therapeutic Considerations

Alone in combination with immunotherapy, anti-angiogenic strategies demonstrated variable efficacy in ES-SCLC. The overall impact is not yet definitive, even though some studies reported high ORR. These approaches may be considered in selected patients or as part of new strategies to be tested in clinical trials. To this concern, it is crucial to identify patient subgroups more likely to benefit from this therapeutic approach, even considering observed treatment-related adverse events.

### 3.6. New Targets for Immunotherapy in Further Lines of Treatment

T-cell immunoreceptor with Ig and ITIM domain (TIGIT) is an immune inhibitory member of the Ig superfamily, with a tyrosine-based inhibitory motif (ITIM) and an immunoglobulin tyrosine tail (ITT)-like motif [113,114]. TIGIT was found expressed on NK, NK-T, CD8+, Tregs and memory CD4+ T cells [115]. While in preclinical models of SCLC, the targeting of TIGIT synergized with PD-1 blockade, reducing tumor growth and prolonging the long-term survival of mice [116,117].

Tiragolumab, a TIGIT inhibitor, received breakthrough therapy designation (BTD) by the FDA, after phase I and II study results in different solid malignancies, including NSCLC [118]. On the other hand, tiragolumab failed to meet primary endpoints in the phase III SKYSCRAPER-02 study, in which the investigation arm in 243 patients tested the addition of tiragolumab to standard atezolizumab plus carboplatin/etoposide of the placebo arm of 247 patients (mPFS 5.4 months, 95%CI: 4.7–5.5 vs. 5.6 months, 95%CI: 5.4–5.9, respectively, and HR_OS_ 1.04, 95%CI: 0.79–1.36, *p* = 0.79). TIGIT targeting is demonstrating efficacy in the AdvanTIG-204 trial, a phase II study comparing the clinical efficacy of ociperlimab (anti-TIGIT) plus or without tislelizumab (anti-PD-1) and plus or without concurrent chemoradiotherapy in limited-stage SCLC [77]. Moreover, the ongoing Keyvibe-008 phase III study is investigating the clinical efficacy of vibostolimab (anti-TIGIT) for chemo-immunotherapy (pembrolizumab or atezolizumab plus carboplatin or cisplatin plus etoposide) [119].

LAG-3 targeting has been attempted also with monoclonal antibodies, apart from bispecific antibodies. The use of ieramilimab (LAG525) alone or in combination with spartalizumab (anti-PD-1) in 75 patients (16 with SCLC) demonstrated acceptable TEAEs rates (56% and 69%, respectively, while serious adverse events were 5% and 5.8%, respectively) [120]. Based on this evidence, the combination of an anti-LAG-3 with an anti-PD-1 could represent a possible strategy to target SCLC and its immune microenvironment, but effective clinical data are needed.

Poly(ADP-ribose) polymerase (PARP) inhibition combined with durvalumab also did not show optimal disease control in subsequent lines of treatment, even though it was tested in a very limited number of patients [121]. In 8 patients receiving lurbinectidin after prior immunotherapy treatment, 5 partial responses and 1 stable disease were observed. PFS values among responders were 8.3, 6.9, 6.3, 7.6 and 4.7 months [122].

Combining multiple immunotherapic drugs in subsequent treatment settings showed potential practice-changing perspectives. Nivolumab and ipilimumab association translated into an ORR of 21.9% and a median duration of response (DOR) of 15.8 months compared with 11.6% and 10 months, respectively, of nivolumab monotherapy. Nevertheless, the higher control rate did not correlate with a benefit in PFS [123]. In the third-line setting, the addition of nivolumab and ipilimumab to rovalpituzumab resulted in a modest improvement in ORR (27.6% to 36.4%) and median OS (7.4 to 11 months), while PFS remained similar (4.8 vs. 4.1 months) [124].

Unfavorable outcomes were observed with the combination of quavonlimab and pembrolizumab, which achieved an ORR of 18% and an mPFS of 2 months [125].

Heterogeneous outcomes from synchronous anti-angiogenic and immunotherapy have been reported. Camrelizumab plus apatinib led to an ORR of 34% and a median PFS of 3.6 months [126]; surufatinib plus toripalimab showed an ORR of 15.8% and a median PFS of 2 months in 20 patients tested after first-line platinum-based chemotherapy [127].

Intensified strategy including also chemotherapy (specifically nabpaclitaxel) administered in second or third line in a small subset of 25 patients guaranteed an ORR of 60% and a median PFS of 6 months [128]. Trials exploring maintenance therapy in patients with SCLC are reported in Table 4.

With regard to chemotherapy-only trials, lurbinectidin plus doxocubicin did not show superiority compared with standard topotecan or CAV in a small subset of IT-pretreated group (mOS 11.4 versus 11.8 months and mPFS 6.9 versus 4.2 months, respectively) [129]. Lack in median OS benefit was observed also when 42 patients received liposomial irinotecan in a phase III trial designed with topotecan in the control arm (mOS: 7.5 and 7.7 months for irinotecan and topotecan, respectively) [130].

The combination of liposomial eribulin and nivolumab in a second line of therapy showed a meaningful ORR of 22.2% and a DCR of 74.1% in a phase II trial but modest median PFS [131].

A high objective response rate (ORR) of 46.4% was observed with the combination of lurbinectedin plus pembrolizumab following first-line platinum-based chemotherapy. Clinical responses correlated with platinum sensitivity, with a median duration of response of 7.8 months and a median progression-free survival of 4.6 months [132].

The results from a phase III trial candidate tarlatamab as standard of treatment after first-line chemotherapy with or without immunotherapy are as follows: The subgroup analysis confirmed a consistent benefit, specifically in patients previously treated with immunotherapic agents. Significant hazard ratios were reported for both PFS (HR_PFS_ 0.61; 95%CI: 0.45–0.82) and OS (HR_OS_ 0.67; 95%CI: 0.53–0.84) in IO-pretreated patients [66]. Key clinical trial results for facing acquired and primary resistance mechanisms to immunotherapy in further lines of treatment are reported in Table 5.

#### Clinical Positioning and Therapeutic Considerations

Emerging immune targets (e.g., TIGIT and LAG-3) and combination immunotherapy approaches have shown mixed results in ES-SCLC. TIGIT and LAG-3 targeting operates at the checkpoint dependency level of the framework (Figure 1). Given the heterogeneity of outcomes and limited definitive survival benefit, these strategies are best pursued in later lines or clinical trial contexts. Dual immune checkpoint blockade, combinations with chemotherapy or anti-angiogenics, and other novel strategies may provide a benefit in selected patients but require further validation.

### 3.7. Resistance to Emerging Therapies (BiTEs/ADCs/Cellular Therapies): Mechanisms and Rationale for Combinations

Despite the encouraging clinical activity of emerging DLL3-targeted therapies, including BiTEs, ADCs, and cell therapies, multiple resistance mechanisms are increasingly recognized. Antigen heterogeneity and antigen loss represent central challenges, particularly in SCLC, a tumor characterized by marked intratumoral and intertumoral heterogeneity. Downregulation or loss of DLL3 expression under therapeutic pressure has been reported as a mechanism of on-target resistance, primarily based on preclinical models, leading to immune escape and disease progression after initial responses to DLL3-directed agents [3,70]. In parallel, lineage plasticity driven by dynamic Notch pathway signaling allows SCLC cells to shift from neuroendocrine to non-neuroendocrine phenotypes, reducing dependency on DLL3 expression and further limiting the durability of targeted therapies [6,7,70].

Resistance to ADCs carrying topoisomerase I inhibitors is also emerging as a relevant issue. Cross-resistance with prior exposure to topoisomerase inhibitors (e.g., topotecan or irinotecan) has been suggested by early clinical observations and preclinical models, potentially mediated by upregulation of drug efflux pumps, enhanced DNA damage repair capacity, and alterations in payload sensitivity, ultimately impairing ADC efficacy [88,94]. These mechanisms raise important questions regarding sequencing strategies and retreatment feasibility with payload-sharing ADCs.

In the context of cellular therapies, antigen density thresholds, heterogeneous target expression, and immune suppressive features of the tumor microenvironment further constrain therapeutic efficacy. Moreover, repeated antigen engagement may promote selective outgrowth of antigen-negative clones, limiting long-term disease control [97,98,99]. Collectively, these observations underscore the need for rational combination strategies, including dual-targeting approaches, epigenetic modulators to stabilize antigen expression, and combinations with immune checkpoint inhibitors or DNA damage response inhibitors, to prevent or overcome resistance. A deeper understanding of resistance biology will be essential to guide patient selection, treatment sequencing, and the design of future combination trials.

### 3.8. Safety-Driven Constraints for Sequencing and Combination Strategies

While emerging therapies have expanded the therapeutic armamentarium for SCLC, safety profiles and feasibility considerations critically influence their optimal sequencing and combinatorial use. Immune-engaging therapies are constrained by the risk of excessive immune activation, as observed across early-phase clinical trials and supported by mechanistic understanding of T-cell redirection. Conversely, ADCs are constrained by cumulative hematologic toxicity and payload-specific cross-resistance, supporting their use in carefully selected later-line settings. Cellular therapies, although conceptually appealing, remain restricted by logistical and safety barriers. Collectively, these constraints highlight that therapeutic sequencing in SCLC is not solely driven by efficacy but must integrate safety-driven feasibility to maximize real-world benefit.

## 4. Discussion

During the last decade, the addition of immunotherapy to standard chemotherapy conferred a survival benefit to patients with SCLC. Nonetheless, the clinical benefit for these patients remains slight, mainly for primary or acquired mechanisms of resistance to immunotherapy.

An immunosuppressive tumor microenvironment, cancer cell adaptation under therapeutic selective pressure, and intratumoral heterogeneity constitute major barriers to effective immunotherapy. Moreover, in the nest of SCLC the cytotoxic T lymphocytes are low in both frequency and density compared with tumor stroma [134], suggesting that SCLC TME acts as a substantial obstacle to cytotoxic cell recruitment, thus impeding the cytotoxic action of effector T cells.

Moreover, anti-cancer immune response is affected by different regulatory processes, mostly guided by T regs, TAMs and MDSCs.

Conversely, in strategies aimed at targeting the TME, reliance on a single biomarker is frequently inadequate to define specific cellular populations (e.g., FOXP3 for regulatory T cells), underscoring the necessity for standardized and multiparametric biomarker approaches.

The combination of different IO-based therapeutic strategies aims to revert the negative immune regulatory mechanisms, resulting in a more effective antitumor effect compared with the use of mono-immunotherapy. Considering that different types of therapy, e.g., cytotoxic agents and radiation therapy, as well as targeted and anti-angiogenic agents, have different anti-cancer effects, they could have different synergistic therapeutic actions when combined with IO.

Based on the evidence summarized in Section 3, strategies combining immune checkpoint inhibitors with cytotoxic chemotherapy or radiotherapy show preliminary activity, but randomized confirmation is lacking. Key uncertainties include optimal sequencing, patient selection, and management of overlapping toxicities. Moreover, new immune-based approaches, such as the engineering of CAR-T cells and the targeting of new biomarkers through precision medicine, could unveil new strategies for immunotherapeutic treatment in SCLC. Rational combination with checkpoint inhibitors or epigenetic modulators could mitigate resistance, but clinical validation is required.

The implementation of transcriptional subtype-driven precision oncology in SCLC remains limited due to several critical barriers, including substantial deep intra-tumoral heterogeneity, not exclusive expression of subtype identifiers biomarkers, and the prognostic relevance of established subtypes.

Other ways to personalize therapies should be explored and further investigated in the future. More effective immune-based combination therapies require further study, such as MHC-I regulation with targeted agents and combination therapies with ICIs. Restoring MHC I expression and enhancing immunogenicity using epigenetic drugs might be a feasible strategy to improve the SCLC TIME and immunotherapeutic effectiveness. Further studies and more in-depth data will likely and hopefully yield greater survival benefits for SCLC patients in the future.

Furthermore, the identification of cost-effective and widely applicable biomarkers is essential for patient stratification and personalized treatment. The influence of finding an optimal cut-off and the variability of different genomic tests limit the potential of TMB as a reliable predictor of immunotherapy benefit [9]. Future efforts should focus on multiparametric biomarker panels integrating TMB, neoantigen clonality, and immune infiltrate features to guide patient selection for immunotherapy, rather than reliance on single markers. In this regard, the effects of intra-tumoral genetic heterogeneity and clonal diversity of malignant cells play a key role for immunotherapeutic treatment as most of the mutations found are often sub-clonal.

Overall, the current therapeutic landscape in SCLC remains challenging. By systematically addressing biological resistance mechanisms, optimizing combinatorial strategies, and standardizing biomarker assessment, the field may achieve more meaningful survival improvements in the coming years.

Resistance to IO remains a hard challenge for clinical management of ES-SCLC, which remains one of the most lethal malignancies worldwide. Looking forward, the integration of molecular subtyping, dynamic profiling of the tumor immune microenvironment, and multi-modal combination therapies—including emerging modalities such as BiTEs and ADCs—may enable truly personalized immunotherapy for SCLC. Harnessing these novel immune-engaging and targeted strategies, together with epigenetic modulation and dual checkpoint blockade, has the potential to transform the current modest benefits into durable clinical responses, ultimately redefining the therapeutic landscape of this aggressive disease.

## 5. Conclusions

SCLC is a highly lethal malignancy, with poor survival. Given the heterogeneity of the malignancy, combination strategies seem to provide the most suggestive results, especially through the new drugs being tested in clinical trials, such as ADCs, bispecific antibodies and adoptive cell therapies.

Further preclinical and clinical studies are needed to address patients to a tailored treatment, to face a malignancy that remains one of the hardest clinical challenges worldwide.

## Figures and Tables

**Table 1:** Subtype-informed therapeutic hypotheses. Possible subtype-oriented therapeutic strategies are presented, based on biological hallmarks and vulnerabilities highlighted by landmark studies. SCLC: small-cell lung cancer; DLL3: Delta-like ligand 3, MHC: major histocompatibility complex; BiTE: bispecific T-cell engager; ADC: antibody–drug conjugates; DDR: DNA damage response; CAR: Chimeric Antigen Receptor; IO: immunotherapy; NE: neuroendocrine; TILs; tumor-infiltrate lymphocytes; ICI: immune checkpoint inhibitors.

SCLC Subtype	Dominant Biology	Immune Phenotype	Hypothesis on Vulnerability	Possible Therapeutic Strategy	Ref.
SCLC-A (ASCL1)	Neuroendocrine-high, DLL3+, MYCL-driven	Immune-cold, low MHC-I, low TILs	DLL3 dependency; Notch pathway dysregulation; epigenetic repression of antigen presentation	DLL3-targeted BiTEs (tarlatamab), DLL3-ADCs, CAR-T/CAR-NK; epigenetic priming + IO	[6]
SCLC-N (NEUROD1)	Neuroendocrine, MYC-driven, aggressive	Immune-cold, DDR-high	Replication stress; MYC-associated immune evasion	DDR inhibitors + IO; DLL3-directed therapies; ADCs	[7]
SCLC-P (POU2F3)	Tuft-cell-like, non-NE	Intermediate immune infiltration	Lineage plasticity; surface antigen expression	ADCs (B7-H3, Trop-2); chemo-IO combinations	[7]
SCLC-I (Inflamed)	Non-NE, interferon-driven	Immune-inflamed, high TILs, higher MHC-I, checkpoint expression	Checkpoint dependency; adaptive immune resistance	ICIs; dual checkpoint blockade; IO + antiangiogenic	[4,7]
Mixed/plastic phenotypes	Dynamic NE ↔ non-NE transitions	Spatially heterogeneous	Lineage switching; antigen loss	Lineage switching; antigen loss	[7]

**Table 3:** Combination strategies in the maintenance setting. In the maintenance setting, different combinations including IO alone and combinations of IO with other agents have been tested to date. Primary endpoints, as well as toxicity and study major limitations, are shown. ES-SCLC: extensive-stage small-cell lung cancer; LS-SCLC: limited-stage small-cell lung cancer; CT: chemotherapy; CT-IO: chemo-immunotherapy; OS: overall survival; PFS: progression-free survival; DCR: disease control rate; TMB: tumor mutation burden; AEs: adverse events.

Experimental Design	Phase	Disease Stage/Setting	Control Arm	Prior IO	Primary Endpoints	Key Results	Toxicity Assessment	Major Limitations	Ref
Nivo + Ipi or Nivo	III	ES-SCLC. Brain mets allowed.	Placebo	No	OS	No OS benefit vs. placebo; signal in TMB ≥ 13 mut/Mb	Immune-related AEs	Negative trial; need biomarker validation	[109]
Nivo + Ipi →Nivo	II	LS-SCLC, maintenance after CT-RT + PCI. Brain mets excluded	Observatory	No	PFS	No PFS or OS benefit	G ≥ 3 AEs 61.5%; 55% discontinuation	High toxicity in curative setting	[110]
Olaparib run-in → durvalumab	I/II	LS/ES-SCLC, platinum-sensitive, maintenance post-CT. Brain mets Allowed.	None	No	Safety/DCR	DCR below pre-specified threshold; mPFS 2.4 mo	Severe hematologic toxicity; treatment-related deaths	Early-phase; unfavorable risk–benefit	[111]
Talazoparib + atezolizumab	II	ES-SCLC, maintenance after 1 L CT-IO. SLFN11+. Brain mets allowed	Atezolizumab	Yes (100%)	PFS	Modest PFS benefit; no OS improvement	G ≥ 3 hematologic AEs 50%	Limited OS impact	[112]

**Table 4:** Combination strategies including radiation therapy. Radiotherapy has been performed in combination with IO or IO + CT as a first or second line, demonstrating encouraging response rates but low benefit in survival rates. ICIs: immune checkpoint inhibitors; RT: radiotherapy; CT: chemotherapy; ES-SCLC: extensive-stage small-cell lung cancer; LS-SCLC: limited-stage small-cell lung cancer; ORR: objective response rate; mOS: median overall survival; mPFS: median progression-free survival; AEs: adverse events; mDOR: median duration of response; TRAEs: treatment-related adverse events.

Therapeutic Class	Experimental Design	Phase	Disease Stage/Setting	Comparator	Primary Endpoints	Key Results	Major Limitations	Ref
ICI + RT	Pembrolizumab q3w + thoracic RT (45 Gy, boost up to 52.5 Gy allowed)	I	Maintenance after induction 1 L CT in ES-SCLC Brain mets allowed.	Single-arm	G3 AEs	G3 Aes 6% mPFS: 6.1 months. mOS: 8.4 months.	Small cohort, no control study	[105]
ICI + RT	Durvalumab + tremelimumab ± SBRT (9 Gy × 3 fx)	II	2 L or ICI-naïve in SCLC Brain mets allowed.	None	ORR	ARM A: ORR 0%,PFS 2.1 months ARM B: ORR 28.6%,PFS 3.3 months	Small cohort, heterogeneous RT schedule	[108]
ICI + RT + CT	Pembrolizumab + concurrent 45 Gy RT + platinum CT	I/II	1 L LS-SCLC (including other neuroendocrine cancers)	None	G4 AEs	3 G4 and 41 G3 events. mPFS19.7 months; mOS: 39.5 months	Small cohort, high rate of ≥G3 AEs	[106]
ICI + RT + CT	Atezolizumab + low-dose thoracic RT (15 Gy/5 fx) + CDDP/CBCDA + etoposide	II	I line for ES-SCLC. Brain mets allowed.	None	ORR	ORR 87.5%; mDOR: 6.9 months. DCR: 94.6%. mPFS: 6.9 months.	High rate of ≥G3 TRAEs, which limit sequencing	[107]

**Table 5:** Strategies in further lines of treatment. New strategies and therapeutic approaches are resumed. Lines of therapy, as well as tumor targets and treatment schedules, are presented; primary and secondary endpoints, as well as comments on study achievements, are reported. ICI: immune checkpoint inhibitor; PARPi: Poly-ADP-ribose polymerase inhibitors; CT: chemotherapy; IO: immunotherapy; BiTE: Bispecific T-cell Engager; CAV: cyclophosphamide, adriamycin (doxorubicin), vincristine; ES-SCLC: extensive-stage small-cell lung cancer; LS-SCLC: limited-stage small-cell lung cancer; ORR: objective response rate; BR: best response; PD: progressive disease; SD: stable disease; DLT: dose limiting toxicity; mDOR: median duration of response; mOS: median overall survival; mPFS: median progression-free survival; DCR: disease control rate; AEs: adverse events; TRAEs: treatment-related adverse events.

Therapeutic Strategy	Phase	Disease Stage/Setting	Experimental Design and Accrual	Line of Therapy	Comparator	Primary Endpoints	Secondary Endpoints	Key Toxicities/ Comments	Ref
ICI + PARPi	II	≥1 platinum-based CT, ES-SCLC. Brain mets allowed	Durvalumab + olaparib. 20 patients (3 pre-IO treated)	≥2 L	Single-arm	ORR: 10.5%	mPFS: 1.8 months.	G ≥ 3 TRAEs: 45% (80% anemia).	[121]
CT	II	ES-SCLC. Brain mets not allowed.	Lurbinectidin. 8/105 patients pre-IO treated	2 L	Single-arm	Response by BR: 5 PR, 2 PD and 1 SD.	PFS; DOR; OS	XX	[122]
ICI + ICI	I/II	LS/ES-SCLC Brain mets allowed	Nivo vs. nivo + ipi (147:96)	≥2 L	Randomized	ORR: 11.6% (N) vs. 21.9% (N + I) cohort.	mDOR; mPFS; mOS Any-G TRAEs N: 53.7%;	N: G ≥ 3 12.9%. N + I: G ≥ 3 37.5%. No OS benefit. Toxicity in combo arm.	[123]
IO + anti-angiogenic	II	ES-SCLC. Brain mets allowed	Camrelizumab plus apatinib. 47 patients	2 L	Single-arm	ORR: 34%	mOS: 8.4 months. mPFS: 3.6 months. DCR: 68.1%.	Single-arm. Vascular toxicity.	[126]
ICI + ICI	I	ES-SCLC. Brain mets allowed	Quavonlimab plus pembrolizumab. 40 patients	2 L	Single-arm	10% reported ≥ 1 DLT. Any-G AEs: 95%, serious AEs 40%.	ORR: 18% mDOR: 12.5 months. mPFS: 2 months. mOS: 11 months.	AE-induced dose modifications 55%.	[125]
ADC + IO	I/II	2 prior CT; ES-SCLC. Brain mets allowed	Rovalpituzumab + nivolumab ± ipilimumab (30:12)	≥2 L	Randomized	1 and 3 DLTs in R + N and R + N + I, respectively.	R + N: ORR 27.6%, mPFS 4.8 and mOS 7.4 months. R + N + I: ORR 36.4%, mPFS 4.1 and mOS 11 months.	G ≥ 3 TRAEs 100% RR + N + I; 87% R + N.	[124]
CT + CT	III	Post-platinum ± IO LS/ES-SCLC. Brain mets allowed	Lurbinectidin plus doxorubicin vs. topotecan or CAV (307:127:179), 6% pre-IO treated	2 L	Topotecan or CAV	mOS: 8.6 (exp) vs. 7.6 (control) months (11.4 vs. 11.8 months in the IO-pretreated population).	mPFS in pre-IO patients: 6.9 vs. 4.2 months in experimental vs. control arms.	TRAEs led to treatment discontinuation in 9% (exp) and 16% (control).	[129]
BiTE	II	2 prior CT. Brain mets allowed	Tarlatamab (10 vs. 100 mg q2w). Pre-IO treated patients: 100 in 10 mg and 62 in 100 mg arms	≥2 L	Randomized	ORR pre-IO treated: 39.7% (10 mg), 32.3% (100 mg). IO-näive: 40.7% (10 mg), 30.8% (100 mg).	DOR, DCR, DDC, PFS, OS, safety.	Formation of tarlatamab-antibodies not explored according to IO-pretreatment.	[64]
CT + IO	II	2 L after platinum ± IO. Brain mets allowed	Liposomial eribulin (E7389-LF) + Nivolumab. 27/33 pre-IO treated	2 L	Single-arm	ORR: 22.2%. DCR: 74.1%	mPFS: 2.86 months. 6-month PFS rate: 21.4%.	XX	[126]
CT	III	2 L after platinum ± IO. Brain mets allowed	Liposomal irinotecan vs. topotecan 42/229 and 43/232 pre-IO treated, respectively	2 L	Randomized	No benefit in OS; pre-IO treatment did not influence OS.	PFS and ORR not explored according to prior IO.	No benefit in OS.	[130]
IO + anti-angiogenic + CT	II	≥2 L ES-SCLC. Brain mets allowed	Sintilimab + anlotinib + nabpaclitaxel. 25 IO-näive patients	≥2 L	Single-arm	ORR: 60%.	mPFS: 6 months; mOS: 13.4 months; mDOR: 5.2 months. DCR: 76%. Any grade TRAE 92%.	G ≥ 3 TRAEs 16% (elevated AST 8%).	[128]
BiTE	I	Previously treated SCLC. Brain mets allowed	Tarlatamab (multiple doses). 84/152 pre-IO treated patients	≥2 L	Dose-randomized	ORR: 25.0%; mDOR: 11.2 months; mOS: 17.5 months.	CNS tumor shrinkage of ≥30% observed in 10/16 of patients with baseline CNS lesion of ≥10 mm.	Lack of mandatory CNS imaging for asymptomatic brain metastasis.	[133]
IO + anti-angiogenic	II	2 L LS/ES-SCLC. Brain mets allowed	Surufatinib + toripalimab. 20 patients	2 L	Single-arm	ORR: 15.8%.	mDOR not achieved. DCR: 94.7%.	Surufatinib-related AEs: 95% (grade ≥ 3 45%). Toripalimab-related AEs: 85% (grade ≥ 3 40%).	[127]
IO + CT	I/II	2 L after platinum	Lurbinectidin + pembrolizumab. 28 patients	2 L	Single-arm	ORR: 46.4% (57.1% Pt-sensitive, 35.7% Pt-resistant cases).	mDOR: 7.8 months; mPFS: 4.6 months; mOS: 10.5 months.	TEAEs 100%, G ≥ 3 TRAEs 71.4%; 17.9% treatment discontinuation.	[132]
BiTE	III	2 L post-platinum	Tarlatamab vs. CT. 180/arm; all pre-IO treated	2 L	Randomized	HR_OS_ 0.61 (95%CI: 0.45–0.82) for IO-pretreated patients.	HR_PFS_ 0.67 (95%CI: 0.53–0.84) for IO-pretreated patients.	First phase III trial positive for BiTE	[66]

## Data Availability

No new data were created or analyzed in this study. Data sharing is not applicable to this article.

## Abbreviations

The following abbreviations are used in this manuscript:

ADC	Antibody–drug conjugate
AE	Adverse event
ATRA	All-trans retinoic acid
B7H3	B7 homolog 3
BiTE	Bispecific T-cell engager
BTD	Breakthrough therapy designation
CAR	Chimeric antigen receptor
CRS	Cytokine release syndrome
CSC	Cancer stem cell
CT	Chemotherapy
DCR	Disease control rate
DDR	DNA damage response
DLL3	Delta-like ligand 3
DOR	Duration of response
EMT	Epithelial–mesenchymal transition
ES-SCLC	Extensive-stage small-cell lung cancer
EZH2	Enhancer of zeste homolog 2
HLA	Human leukocyte antigen
HMGA1	High-Mobility Group AT-hook 1
ICI	Immune checkpoint inhibitor
I-DXd	Ifinatamab–deruxtecan
IO	Immunotherapy
iPSC	Induced pluripotent stem cell
ITIM	Tyrosine-based inhibitory motif
ITT	Immunoglobulin tyrosine tail
LAG-3	Lymphocyte activation gene-3
LSD1	Lysine-specific methylase 1
mAb	Monoclonal antibody
MDSC	Myeloid-derived suppressor cell
MHC	Major histocompatibility complex
NE	Neuroendocrine
NK	Natural killer
NSCLC	Non-small-cell lung cancer
ORR	Objective response rate
OS	Overall survival
OS	Median overall survival
PARP	Poly-ADP-ribose polymerase
PD-1	Programmed Death 1
PD-L1	Programmed death-ligand 1
PFS	Progression-free survival
mPFS	Median progression-free survival
Rova-T	Rovalpituzumab terisine
RT	Radiotherapy
SBRT	Stereotactic body radiation therapy
scFv	Single chain variable fragment
SCLC	Small-cell lung cancer
SEZ6	Seizure-related homolog 6
SG	Sacituzumab govitecan
TAM	Tumor-associated macrophage
TCR	T-cell receptor
T regs	Regulatory T cells
TEAE	Treatment emergent adverse event
TIGIT	T-cell immunoreceptor with Ig and ITIM domain
TIL	Tumor immune lymphocyte
TIME	Tumor immune microenvironment
TMB	Tumor mutation burden
TME	Tumor microenvironment
TRAE	Treatment-related adverse event
Treg	Immune regulatory T
TriTAC	Tri-specific T-cells activating constructs
Trop-2	Trophoblast cell surface antigen 2
VEGF	Vascular endothelial growth factor
